# Supplementary material for: Understanding digital health technology implementation in rehabilitation and development of the Rehabilitation Technologies Implementation model
Source: NPJ Digit Med. 2026 Apr 4;9:421. doi: 10.1038/s41746-026-02599-1 (PMC13230725; doi:10.1038/s41746-026-02599-1)
Supplement: Supplementary file 2 — Supplementary material_Table 1. [file 41746_2026_2599_MOESM2_ESM.docx]

**SUPPLEMENTARY MATERIAL**

Supplementary Table 1 – Alignment between domains of the RiTe model and other models with indicative quotes from qualitative interviews.

| Domain | Constructs | Features | Alignment with key models | Indicative quotes |
| --- | --- | --- | --- | --- |
| **Evidence** | Evidence | Evaluating the available evidence to inform technology selection | NASSS (wider system & value proposition), e-HIT (Context & Intervention) DALLAS (national policies), Sociotechnical transition theory (Sociotechnical system), Health Technology evaluation framework, ARCHIE (evaluation). | “So what's the evidence behind it? And I guess, what's the theory behind it?” P31 (Clinician)  “It produces outcomes we want; it appeals to the client group we deal with and, and the therapist seem to like it as well” P30 (Service manager) |
|  | Evaluation | Identification and rigorous measurement of the key outcomes that can be used to judge the success of the implementation process and evaluate its use and effectiveness of the technology in the service. | CFIR (Innovation outcomes & Implementation outcomes), e-HIT (Intervention), TAM2 (demonstrability), NASSS (embedding &evaluation over time), MRC (mechanisms of impact & outcomes), Social Practice Theory (Connections), Normalisation Process Theory (reflexive monitoring), ReAIM, PDSA, DHEquiR (unmet needs), Knowledge to Action, Health Technology evaluation framework. | “There needs to be good data to show that it's it works for the patient and….the clinician” P41 (Clinician)  “...is this really acceptable for patients, is this a beneficial way to do it?” P4 (Service manager) |
| **Technology** | Ease of use | The physical, technical and simplicity of initially setting up, and then using the technology regularly. | e-HIT (Intervention - ease of use), TAM2 (perceived ease of use), NASSS (technology) CFIR (innovation design & innovation complexity), Health Technology evaluation framework, DEDHI (Interoperability), | “It was a really plain and simple app to use it… it wasn't complicated, it basically it would lead you” P103 (Service user)  “That connected me up to it and it was straight forward. It wasn't complicated to do.” P50 (Service user)  “It was very clunky. It didn't work very well. So we had to kind of try and work around how to make it work within what we were told we could do the restrictions of the software and the people that were programming it, who weren't clinical.” P10 (Clinician and Entrepreneur) |
|  | Features of the technology | The suitability of the technology for its intended use including benefits and alignment with existing workflows. | NASSS (patient condition, technology, value proposition), TAM2 (performs required task) DHEquiR (target population), Normalisation Process Theory (coherence), Motivational theory/design, sociotechnical change model. | “We thought [the technology] captured most of our patient population to get most use out of [it].” P22 (Clinician)  “This is definitely going to help support them. I couldn't really pinpoint the degree to which it would help them, but I knew that it was going to help in some type of way” P33 (Support staff) |
|  | Features of the environment | Consideration of the intended environment the technology is to be used in. This includes physical factors such as space for equipment but also connectivity, hardware etc required. | DALLAS (Infrastructure & Interoperability), Social Practice Theory (materials), CFIR (space, physical infrastructure) | “I think portability as well is quite handy if because we operate in two…different rooms” P33 (Support staff)  “We always have a bit of a problem kind of finding space where to use it…cause it's also not got its own space. So you have to make sure no one else is in the one space it can be used in.” P25 (Clinician) |
|  | Factors influencing service user access | Real-world, physical factors that influence who can access and utilise the technology. | DHEquiR (strategies/barriers), UTAUT, TAM2 (subjective norm), Social Construction of Technology (relevant social group), ARCHIE (human), NASSS (adopters), DALLAS (patients), e-HIT (resources) | “But I know that there's so many of them out there that can't access it for so many different reasons, and they're getting left behind. And it's all very well doing all this digital or whatever…. What about all the people are getting left behind?” P13 (Clinician)  “Because we're rural, the internet connections can be a bit dodgy” P43 (Innovation lead)  “[Service users] shouldn't be restricted from using the tablet because they haven't got Wi-Fi” P80 (Information technology specialist) |
|  | Funding (pre implementation | The process of identifying funding and the processes to utilise funding to purchase the technology and maintain it. | CFIR (Innovation cost, funding), NASSS (organisation), e-HIT (resources and risk), DEDHI (cost, reimbursement) | “I suppose it was like a shortened business case that had to do with more outlining the benefits for our patients and the business case and the cost implications.” P71 (Service lead)  “So you buy … a hundred, 200, £300,000 worth of equipment and then you don't have the funding for maintenance.” P21 (Information technology specialist). |
|  | Selecting the ‘right’ technology (pre-implementation) | Understanding the available technologies on the market and their selection. | CFIR (Innovation relative-advantage & Innovation trialability), NASSS (value proposition), Grol’s Implementation model (economic & political), Normalisation Process Theory (coherence), DALLAS (market), Health Technology evaluation framework (Input). | “So it wasn’t suitable so we had to find an alternative platform” P4 (Service manger)  “The technology itself and the fact that it was medically labelled (as) one was pretty much irrelevant. I expect it was 100 times more expensive and it was 1/5 of the feature set that they got if you'd just gone to *technology Y*.” P105 (Service user) |
| **Users** | Service users | The beliefs, experiences and needs of service users (and those that care for them) to access and utilise the technology. | Theory of planned behaviour (attitude, perceived behavioural control), DALLAS (agency, digital literacy), Behaviour Change wheel, COM-B (capability, motivation), Motivational theory/design, DHEquiR (disadvantaged groups, quantify inequities, training & support) DEDHI (equity & ethics), Normalisation Process Theory (cognitive participation & collective action), Grol’s Implementation model (individual patient), CFIR (individuals), Theory of planned behaviour (attitude), Theory of reasoned action (beliefs and behaviour), Bowen’s feasibility framework (acceptability, practicality). ARCHIE (human). | “I just got to the point where I just thought it's too much trouble …you almost kind of think it's putting more pressure on me than I can you know the that I can benefit from this sort of thing” P54 (Service user)  “I mean it's easier, it was definitely easier for me because I don't drive and I didn't have to go anywhere so that's kind of easier in one sense. But in the other sense, I didn't know if I was doing them right.” P52 (Service user)  “When I actually went into it, … once the baseline had been set, I did one, I did 2 sessions and I just thought what's the point? This is so easy. It's Noddy stuff. At that stage, because it was learning, it took me into the next level. It's then I realised hang on a minute…. I'm struggling here I actually can't do this, so it was probably about 3 sessions online in that I kind of realised, actually this is this is hard, this is tough.” P104 (Service user) |
|  | Staff users | The beliefs, experiences and needs of staff users to use the technology as part of their usual practice. | Theory of reasoned action (subjective norms), theory of interpersonal behaviour (expectations, values and emotions), Theory of planned behaviour (attitude, perceived behavioural control), DALLAS (agency, digital literacy), Behaviour Change wheel, COM-B (capability, motivation), Motivational theory/design, Theoretical Domains Framework (professional role), TAM2 (perceived usefulness), NASSS (adopters), CFIR (Individuals), Social construction of technology (social group), Social cognitive theory (behavioural and environmental factors), Normalisation Process Theory (cognitive participation & collective action, Social Cognitive learning Theory (cognitive factors). | “I think it was easy because the staff were on board with it because they knew there was benefit for patients. I think that was the biggest thing that made it easier to bring it into place”. P71 (Service manager)  “I think that's about people's own confidence working with technology and understanding technology and also their own interest and motivation for it as well.” P30 (Clinician) |
|  | - *Resources and planning* | Recognition that implementation of technology requires obvious (e.g. funding) and hidden resources (e.g. time) and technology implementation will change models of current service provision. | e-HIT (resource & risk), Behaviour Change wheel, COM-B (opportunity), NASSS (organisation), DHEquiR, CFIR (Innovation cost, funding), Health Technology evaluation framework (Input). | “Everybody's so busy …. they don't have time” P12 (Clinician)  “I think there's a lot of operational things which maybe haven't been thought through in enough detail to make it work, but probably could be if somebody put the effort into making it work.” P10 (Clinician and Entrepreneur)  “So particularly at the beginning when it's more time consuming, when no one knows how to use it and it's hard to set up and the familiarity isn't there, you know the lack of time makes the, you know, means that it takes longer to implement than it should”. P20 (Clinician) |
|  | - *Willingness to use* | Understanding of the evidence underpinning a technology, beliefs and values of the benefit offered by the technology, plus staff users confidence to implement and use the technology. This includes staff interest in technology and their current ability to embrace change to their daily practice. | Behaviour Change wheel, COM-B (opportunity, motivation), Diffusion of Innovation, TAM2 (relevance, performs required tasks, voluntariness), Social Practice Theory (cognitive), DALLAS (readiness), NASSS (adopters), Normalisation Process Theory (cognitive participation), e-HIT, (workforce, impact on workflow), Motivational theory/design, DEDHI (acceptability, workflow), Grol’s Implementation model (individual professional), Theory of interpersonal behaviour (expectations), CFIR (individuals) Theory of planned behaviour (attitude) Bowen’s feasibility framework (acceptability), Theory of reasoned action (beliefs and behaviour) | “I think it is just purely motivated by, okay, what can we use as a tool to improve people's outcomes, which is what it's all about.” P31 (Clinician)  “It's just people's personality types that some people are very risk averse and they don't want to do something different and maybe they'll do it when they see it in a journal and all their friends are doing it. Maybe then they'll think ‘ohh maybe we could do that’. But they don't want to be the first one doing it.” P10 (Clinician and Entrepreneur) |
|  | - *Support from technology providers* | The provision of timely, tailored support from technology providers to enable implementation, continued use, training and troubleshooting. | DHEquiR (training & support, resources & incentives),NASSS (technology), TAM2 (results demonstrability, experiences, voluntariness), Behaviour Change wheel, COM-B (opportunity – social support) | “I think that was the biggest thing that made it easier to bring it into place. I think the support from the company as well made it easier- if you know that there's always someone there to kind of go back to if you have any difficulties.” P71 (Clinician)  “It takes forever to get a technician to come out and sort something, and that that would then have an impact. You know if it takes five days for someone to come and check something out, that's five days of rehab that someone's not getting, that's five days lost of strengthening.” P23 (Clinician) |
|  | - *Learning and support from other teams/groups within or outside the organisation* | Use of learning from others who have implemented or used the technology. | Social Cognitive learning Theory (learning from each other), NASSS (wider system). Behaviour Change wheel, COM-B (opportunity – social support) | “It's good to be in touch with and attend those I think that you have those meetings at NEC Birmingham and at Excel where they have all the kit. And just go and see what's happening in different units.” P24 (Clinician) |
|  | - *Training and skills* | The training and skills required so that staff are competent and confident to utilise specific technology and to support others to use it. | Social Practice Theory (competencies), NASSS (technology), e-HIT (resources, education & training), Behaviour Change wheel, COM-B (capability), DALLAS (training, confidence and readiness), Social learning theory, Social-cognitive theory (self efficacy, learning from each other), Theory of planned behaviour (perceived behavioural control) | “The staff training, for them to feel confident enough to actually utilise it” P22 (Clinician)  “I suppose the main difficulty is the time it takes for the training of the staff is one of the difficulties I suppose, and it's not something you can just do once, you need to keep like, going back to it on repeating it and making sure.” P71 (Service manager) |
| **Team** | - Staff champions | An identified individual or group from within the team who will lead and support the technology implementation. | Social Learning Theory, DH Equi R (key actors) Social cognitive learning theory (vicarious learning, modelling), e-HIT (local person, local culture & sponsor), TAM2 (subjective norm, peer perception), Theory of reasoned action (subjective norms), Theory of interpersonal behaviour (social factors), CFIR (Individuals – opinion leaders, implementation members), UTAUT (social influence), Social construction of technology (relevant social group) | “But I think the key person that's been driving it forward has been doing an excellent job of implementing it and getting it embedded into the unit.” P20 (Clinician)  “However you dress it up, they need to be positive about it, to keep talking about it, keep bringing it up, to lead by example. And to, you know, to kind of like gently ask questions in terms of, ‘OK, if not now, when?’” P40 (Clinician) |
|  | - Team characteristics | The culture, beliefs and behaviours of the team who will be using the implemented technology | CFIR (Individuals – opinion leaders, implementation members), Social Learning Theory, DH Equi R (key actors), Theory of interpersonal behaviour (social factors), social construction of technology (relevant social group). | “Our teams very proactive in implementing new things and always looking to how they can improve the service. So they are very positive whenever training to implement something new, they're always on board with it and happy to trial all sorts of different things.” P71 (Service manager) |
|  | - Local leadership | This is local manager or team leader who has oversight for the day-to-day running of the team/service | e-HIT (opinion leader & sponsor), sociotechnical change model, NASSS (Organisation), social construction of technology (technology frame), CFIR (inner setting) | “I would say absolutely leadership engagement, from a clinical perspective. The clinical lead was an absolute pivotal to it being implemented from both her relationships upwards within the network and within the management level but also to the therapy staff on the ground and giving people permission to be involved.” P1 (Clinical researcher) |
|  | - Strategic leadership | This is an organisational leader who has insight and (some) influence upon the strategic direction and priorities of the service and wider organisation. | e-HIT (opinion leader & sponsor), DH Equi R (key actors), CFIR (inner and outer setting), UTAUT (facilitating conditions) | Having the executive sponsorship was essential particularly for this organization of this size and complexity, (be) cause that signalled this is important. P32 (Clinician) |
| **Organisation** | - Support | Sources of support for technology implementation that exist within the organisations. | e-HIT (opinion leader & sponsor), sociotechnical change model, NASSS (Organisation), social construction of technology (technology frame), CFIR (inner setting) | “…that was benefit of think of having one foot in digital health already because I kind of knew these people and I was in the meetings and I could. Yeah, I could get to the right people because I was working with them.” P10 (Clinician and Entrepreneur) |
|  | - Readiness and priorities | The current ability and willingness of the organisations to implement technology. | Socio-technical system (readiness & priorities), UTAUT (facilitating conditions), NASSS (organisation), CFIR (outer setting). | “There's a lot about developing the culture for innovation” P81 (Service manager)  “It'll be the blocks every way you turn, you'll get a block, and if you don't, just keep going on and on and on and on about it people don't reply and then you have to keep following it up and following it up …” P82 (Clinician) |
|  | - Characteristics | The attitude towards, and previous experience of, implementing (technological) change within the organisations. | NASSS (organisational resilience), Socio-technical system (culture & policy), DALLAS (organisational culture), iPARIHS (organisation), Theory of planned behaviour (subjective norm), sociotechnical change model. | “It's almost as though technology snuck up on us and it's brand new, arrived last week, and nobody knows what to do about it.” P31 (Clinician)  “There is no sort of coordinated drive within the organisations to implement things. It's often individual interest and drive of individual clinicians who pick up things and then try and implement something and quite often are crushed by the process of going through it” P22 (Clinician) |
|  | - Operational processes and permissions | Understanding of the operational processes and permissions that must be completed to implement a new technology. | Sociotechnical system (policy), NASSS (technology, wider system & value proposition), CFIR (local conditions), Grol’s implementation model (organisation context), Normalisation process theory (collective action), DALLAS (resources), MRC (context), iPARIHS (innovation), UTAUT (facilitating conditions), DEDHI (safety) | “We can all be really passionate about it and wanting to implement it, but it doesn't just fall on us - we can sing and dance as we want. But there's a whole system involved in getting this implemented” P25 (Clinician) |
|  | - IT and IG involvement | The Information technology and governance scrutiny required before implementation of a new technology or addition of new features to an existing technology | DALLAS – (local readiness & IT infrastructure), e-HIT (context), Sociotechnical system (policy), DEDHI (safety), NASSS (organisation & wider system), DH EquiR (outcomes). | “We have good relationships with our IT team” P61 (Clinician)  “Know your IT processes, from day dot to stop any time wasting.” P70 (Clinician)  “Then there's lots of phone calls and then IT don't answer you…I was getting ignored at my level - at Band 7 level. I had to go right up to the therapy lead in the trust and copy her into my emails, and then all of a sudden I was on a call with the IT manager and things started moving” P82 (Clinician) |

Abbreviations: DHEquiR – Digital Health Equity-focused Implementation Research Model, DEDHI – Design and evaluation of Digital health interventions, CFIR – Consolidated Framework for Implementation Research, e-HIT – e-Health Implementation Toolkit, NASSS – Non-Adoption, Abandonment, Scale-up, Spread and Sustainability framework, iPARIHS - integrated-Promoting Action on Research Implementation in Health Services framework, TAM2 – Technology Acceptance Model 2, UTAUT – Unified Theory of Acceptance and Use of Technology.
